# EP05 COVID-19 and the biliary tree: atypical manifestations of disease during the pandemic

**DOI:** 10.1093/rap/rkaa052.004

**Published:** 2020-11-03

**Authors:** Thomas Williams, Elizabeth Price, Azeem Ahmed

**Affiliations:** Great Western Hospital NHS Foundation Trust, Swindon, United Kingdom

## Abstract

**Case report - Introduction:**

COVID-19, the infectious disease caused by the novel coronavirus SARS-CoV-2, and first described in Wuhan, China in December 2019, has affected more than 19 million patients worldwide and resulted in more than 700,000 deaths at the time of writing^1^. Patients with rheumatic diseases and those receiving immunosuppressive treatment are felt to be at greater risk of complications from this illness, though registry and trial data should help refine our understanding of these risks. We hereby describe a case of COVID-19 complicating an unusual rheumatic illness, resulting in severe multi-system disease and premature death.

**Case report - Case description:**

A 69 year-old male presented to rheumatology and haematology with symmetrical polyarthritis, thrombocytopenia (18 x 10^9^/L), eosinophilia (25.4 x 10^9^/L), raised C-reactive protein (CRP, 43 mg/L), positive rheumatoid factor (>200), antinuclear antibody (ANA) and anti-Ro. Bone marrow biopsy did not demonstrate evidence of haematological malignancy.

Seropositive rheumatoid arthritis and connective tissue disease overlap were diagnosed, and treatment with Prednisolone 60mg daily was initiated. Despite rituximab and intravenous immunoglobulins, thrombocytopenia deteriorated on reducing corticosteroids, however the addition of mycophenolate mofetil (MMF) allowed gradual prednisolone tapering to 3mg daily. Hydroxychloroquine was briefly added but discontinued due to headaches. MMF was discontinued after he developed fungal pneumonia followed by jaundice. Liver biopsy was consistent with drug-induced cholestasis, attributed to co-amoxiclav, and his liver function tests (LFTs) improved on ursodeoxycholic acid. Following a further deterioration in thrombocytopenia, hyperferritinaemia and new onset erythema nodosum, he had a repeat bone marrow examination. This demonstrated large areas of fibrosis and granulomatous inflammation with a dense, pleomorphic T-cell infiltrate, but no haemophagocytosis. Haematologists felt this was reactive and prednisolone dose was increased to 10mg daily.

Six months later he developed cholangitis. Magnetic resonance cholangiopancreatography (MRCP) demonstrated a tight 4cm stricture of the distal common bile duct (CBD) within the head of pancreas, which was diffusely swollen without any clear focal mass. Serum amylase was mildly elevated (316 units/L). Concurrent CT thorax, abdomen and pelvis demonstrated bilateral ground-glass changes within the lungs, and a SARS-CoV-2 nasopharyngeal PCR test was positive, though he had no respiratory symptoms or oxygen requirement at that stage.

Sadly, four days after the CT scan and before a planned endoscopic retrograde cholangiopancreatography (ERCP) could be performed, he became markedly hypoxic with plain chest x-ray features suggestive of COVID-19 pneumonia. Despite medical management, including doubling of his prednisolone dose, he rapidly deteriorated and died.

**Case report - Discussion:**

This case highlights an unusual presentation of COVID-19 in a patient with a complex background of inflammatory arthritis with immune-mediated thrombocytopenia. At the time of his final illness, these conditions were managed with steroid monotherapy. Based on the COVID-19 risk matrix recommended by the British Society for Rheumatology, he was not identified as a patient requiring shielding.

Cholangitis was the major problem precipitating his final admission to hospital, and at the time of admission he had no respiratory symptoms. One week prior to this admission, his father-in-law had died of COVID-19 pneumonia, though they had not been in recent direct contact. Interstitial lung changes were incidentally noted on a CT performed to identify the cause of cholangitis, which prompted the nasopharyngeal PCR that detected SARS-CoV-2. This occurred prior to widespread routine testing of hospital inpatients for SARS-CoV-2 by PCR. Unfortunately he then rapidly developed COVID-19 pneumonia and died before the underlying cause of cholangitis could be definitively identified, though an MRCP demonstrated an obstructed CBD within a diffusely swollen pancreas, where a differential diagnosis of pancreatic malignancy or autoimmune pancreatitis was suggested by the reporting radiologist.

There are emerging case reports of COVID-19 resulting in significant pancreatic injuryand a further recent laboratory analysis has suggested that ACE2 receptors, which are utilised by SARS-CoV-2 to gain entry to host cells, are highly expressed on cholangiocytes at a comparable level to type II alveolar cells. Whilst the ultimate cause of cholangitis will remain unknown in this patient, this case highlights the potential for atypical presentations and extra-pulmonary manifestations of COVID-19.

**Case report - Key learning points**
 

1. COVID-19 is a multi-system illness which can cause significant extra-pulmonary as well as pulmonary pathology, with emerging reports that the biliary tract and pancreas are frequently affected.
2. Evidence to inform accurate prediction of which patients with rheumatic diseases are at highest risk of acquiring severe COVID-19 disease remains insufficient, with current shielding guidelines based on expert consensus.
3. This case highlights the importance of widespread testing for COVID-19 in hospital patients, as not all patients carrying the SARS-CoV-2 virus will demonstrate classical respiratory features of the disease at the point of admission.

